# The Role of Predictive Models in the Assessment of the Poor Outcomes in Pediatric Acute Liver Failure

**DOI:** 10.3390/jcm11020432

**Published:** 2022-01-15

**Authors:** Tudor Lucian Pop, Cornel Olimpiu Aldea, Dan Delean, Bogdan Bulata, Dora Boghiţoiu, Daniela Păcurar, Coriolan Emil Ulmeanu, Alina Grama

**Affiliations:** 1 2nd Pediatric Discipline, Department of Mother and Child, Iuliu Hatieganu University of Medicine and Pharmacy, 400012 Cluj-Napoca, Romania; gramaalina16@yahoo.com; 2 Center of Expertise in Pediatric Liver Rare Disorders, 2nd Pediatric Clinic, Emergency Clinical Hospital for Children, 400177 Cluj-Napoca, Romania; 3 Pediatric Nephrology, Dialysis and Toxicology Clinic, Emergency Clinical Hospital for Children, 400177 Cluj-Napoca, Romania; cornelaldea65@yahoo.com (C.O.A.); ddelean2003@yahoo.com (D.D.); iggbie@gmail.com (B.B.); 4 Department of Pediatrics, Carol Davila University of Medicine and Pharmacy, 020021 Bucharest, Romania; dora.boghitoiu@umfcd.ro (D.B.); daniela.pacurar@umfcd.ro (D.P.); coriolanulmeanu@yahoo.com (C.E.U.); 5 Department of Pediatrics, Grigore Alexandrescu Emergency Clinical Hospital for Children, 011743 Bucharest, Romania

**Keywords:** acute liver failure, children, prognosis, pediatric end-stage liver model, model for end-stage liver disorder, King’s College Hospital criteria

## Abstract

Objectives: In children, acute liver failure (ALF) is a severe condition with high mortality. As some patients need liver transplantation (LT), it is essential to predict the fatal evolution and to refer them early for LT if needed. Our study aimed to evaluate the prognostic criteria and scores for assessing the outcome in children with ALF. Methods: Data of 161 children with ALF (54.66% female, mean age 7.66 ± 6.18 years) were analyzed based on final evolution (32.91% with fatal evolution or LT) and etiology. We calculated on the first day of hospitalization the PELD score (109 children), MELD, and MELD-Na score (52 children), and King’s College Criteria (KCC) for all patients. The Nazer prognostic index and Wilson index for predicting mortality were calculated for nine patients with ALF in Wilson’s disease (WD). Results: PELD, MELD, and MELD-Na scores were significantly higher in patients with fatal evolution (21.04 ± 13.28 vs. 13.99 ± 10.07, *p* = 0.0023; 36.20 ± 19.51 vs. 20.08 ± 8.57, *p* < 0.0001; and 33.07 ± 8.29 vs. 20.08 ± 8.47, *p* < 0.0001, respectively). Moreover, age, bilirubin, albumin, INR, and hemoglobin significantly differed in children with fatal evolution. Function to etiology, PELD, MELD, MELD-Na, and KCC accurately predicted fatal evolution in toxic ALF (25.33 vs. 9.90, *p* = 0.0032; 37.29 vs. 18.79, *p* < 0.0001; 34.29 vs. 19.24, *p* = 0.0002, respectively; with positive predicting value 100%, negative predicting value 88.52%, and accuracy 89.23% for King’s College criteria). The Wilson index for predicting mortality had an excellent predictive strength (100% sensibility and specificity), better than the Nazer prognostic index. Conclusions: Prognostic scores may be used to predict the fatal evolution of ALF in children in correlation with other parameters or criteria. Early estimation of the outcome of ALF is essential, mainly in countries where emergency LT is problematic, as the transfer to a specialized center could be delayed, affecting survival chances.

## 1. Introduction

In children, acute liver failure (ALF) is a rare but severe disorder associated with high mortality [1,2]. Pediatric ALF (PALF) differs from adults due to the type and diversity of causes and late appearance of hepatic encephalopathy (HE) [1]. The etiology of ALF in children varies according to age and worldwide location [3,4,5,6,7]. Children with autoimmune hepatitis (AIH), hepatitis A virus (HAV) infection, and acetaminophen overdose are more likely to survive without liver transplantation (LT). On the contrary, neonates with Herpes simplex virus (HSV) infection and ALF have 10% recovery chances only with antiviral treatment [8,9]. In PALF, 20% of those who never developed HE died or underwent LT, and those with grade IV HE had a better outcome than those who progressed to grade IV [10]. During the last decades, spontaneous survival in patients with ALF increased from 17 to 48% [11]. Moreover, survival after LT increased from 10–56 to 60–86% [11,12,13,14].

Nowadays, 10–15% of LT indications in children are in ALF patients [3,15]. It is difficult to decide to transplant a child with ALF as it is impossible to know if the patient may survive without LT [16]. LT would offer a chance of survival to the high-risk ALF patients, but some patients would be at risk of unnecessary surgery and lifelong immunosuppression. Successful conservative management would save low-risk ALF patients from unnecessary surgery [11,12,17].

Accurate prognostic scores are needed to discriminate patients with poor outcomes from those who will recover spontaneously or with a specific treatment [3,12,18,19]. More and more, there is a need to optimize the available organs and resources [3]. The moment of the listing is critical for the success of LT in patients with ALF [12]. The availability of organs or living donors dictates when a patient with pediatric ALF is transplanted once the patient is listed, and varies based on a center experience with different LT types. Moreover, some patients with LT would survive without it [20].

Researchers developed, evaluated, and revised scoring systems to aid the prediction of the ALF outcome. An accurate model would require a large cohort and a multicenter approach [16]. The predictive models are based on features derived from analysis of ALF cohorts, treated conservatively, without LT [21]. Until now, many factors proved to be possible prognostic markers for an unfavorable outcome in pediatric ALF: age under ten years, etiology (viral infections, Wilson’s disease—WD), the presence of grade III/IV HE, a quick decrease in the liver volume or serum level of aminotransferases, increased level of bilirubin, severe coagulopathy (high International Normalized Ratio, INR, and decreased level of coagulation factors V and VII). Moreover, alpha-fetoprotein (AFP), serum phosphate, serum lactate, ammonia, vitamin D-binding protein were identified as possible prognostic factors in ALF [3,15,22,23,24,25,26,27].

There is no predictive model universally accepted for evaluating ALF outcomes in children [1,28], and all are based upon data and experiences in adults [10]. In adults, the most used models are the King’s College Criteria (KCC), Clichy criteria, Model for End-stage Liver Disease (MELD), and Wilson index for predicting mortality in WD. The Pediatric End-stage Liver disease (PELD) model was evaluated in children. Models used in Intensive Care Units (ICU) were also analyzed [1,29,30]. There is an urgent need for further research in a dynamic scoring system that should include etiology and age [1]. Prompt referral to a transplant center is crucial for those with high risk, and ideally, the prognostic scores should be based on easy to obtain biochemical parameters and clinical features [8]. An ideal predictive model in ALF will help us to be sure that all children needing an LT will receive it (higher sensitivity and positive predictive value, PPV), and those who would survive without LT would not have it (higher specificity and negative predictive value, NPV) [1,19,31].

Our study aimed to analyze PELD/MELD scores and KCC’s role in predicting the survival with native liver in children with ALF of different etiologies. Moreover, we analyzed the Nazer prognostic index and Wilson index for predicting mortality performance in WD patients.

## 2. Materials and Methods

We analyzed retrospectively the children (0 to 18 years of age) hospitalized for ALF in the two leading children’s hospitals in ALF management in Romania (Emergency Clinical Hospital for Children Cluj-Napoca and Grigore Alexandrescu Emergency Clinical Hospital for Children Bucharest), between 2012 and 2018. There were 161 children diagnosed with ALF whose medical charts were analyzed, recording their demographic data, and clinical and laboratory parameters.

The study was conducted according to the guidelines of the Declaration of Helsinki, and it was approved by the Ethics Committee. Informed consent was obtained from all subjects involved in the study.

The diagnosis of ALF followed the PALF study group criteria: biochemical evidence of acute liver injury, hepatic-based coagulopathy (prothrombin time, PT > 15 s, INR > 1.5 not corrected by vitamin K in the presence of HE; or PT > 20 s or INR > 2.0 regardless the presence of HE) in a patient with no evidence of previous chronic liver disease [10].

Our patients were analyzed function to ALF evolution: poor outcome, defined as LT or death, and good outcomes, defined as long-term survival with native liver (no LT). Based on the age, we grouped our cohort in neonates and infants (under 12-month-old), children (1–12-year-old), and teenagers (12–18-year-old).

We included all patients under 18 years of age, with ALF diagnosis based on PALF criteria, and complete clinical and laboratory data. We excluded the patients that did not fulfill PALF criteria and those without all data needed for MELD or PELD scores calculation or KCC evaluation. We also excluded the patients with ALF due to acute infection with HAV or hepatitis E virus (HEV) hospitalized at Infectious Disease Hospitals based on local epidemiological rules and patients with ALF in multiple systems organ failure (MSOF), trauma, burns, or oncological disease after chemotherapy.

We collected the following data from the patients’ medical charts: demographic data (age, sex), ALF etiology (infectious, metabolic, toxic-mushroom poisoning or drug-induced, autoimmune, and unidentified), and laboratory results from the first day of hospitalization (alanine aminotransferase, ALT, aspartate aminotransferase, AST, total bilirubin, TB and direct bilirubin, DB, PT, INR, serum albumin, sodium, creatinine, white blood cells, WBC, hemoglobin, and platelets). For the etiology of ALF, we have used the laboratory tests according to the diagnostic protocols. In ALF due to infections, we analyzed AgHBs for HBV, specific antibodies or viral load for hepatitis C virus (HCV), Cytomegalovirus (CMV), Epstein–Barr virus (EBV), HSV or enteroviral infection, specific antibodies for Leptospira, inflammation markers (erythrocyte sedimentation rate, ESR, C reactive protein, CRP, and procalcitonin), and blood cultures for bacterial infections. In the metabolic causes of ALF, we analyzed specific enzyme tests, serum amino acids, urinary organic acids, and serum or urinary metabolites evaluated by Nuclear Magnetic Resonance (NMR) spectroscopy [32,33]—galactose, galactitol, tyrosine, and succinyl-acetone. Moreover, genetic tests or histopathologic exams were used to diagnose inborn errors of metabolism (IEM). We have assessed the serum ceruloplasmin and copper level and the 24 h urinary copper excretion for WD diagnosis. For autoimmune etiology of ALF, we analyzed immunologic tests: serum level of IgG, antinuclear antibodies (ANA, ≥1:40), anti-smooth muscle antibodies (anti-SMA, ≥1:20), anti-soluble liver antigen antibodies (anti-SLA), anti-liver kidney microsomal type 1 (anti-LKM-1, ≥1:20), or anti-liver cytosol antibodies (anti-LC1). The toxic-induced liver injury was diagnosed based on a mushroom or drug ingestion’s positive history after other ALF causes were excluded or using urinary or blood toxicology exams.

For every patient, we calculated PELD (in children under 12 years old), MELD, and MELD-Na score (in teenagers) on the first day (admission day), using an online calculator (https://www.mdcalc.com, last accessed 12 December 2021), based on formulas previously described: PELD = 0.436 (age (<1 year))-0.687 log (serum albumin g/dL) + 0.480 × log (TB mg/dL) + 1.857 log (INR) + 0.667 (growth retardation (<−2 SD)) [34], MELD = 3.78 × log[TB (mg/dL)] + 11.2 × log[INR] + 9.57 × log[serum creatinine (mg/dL)] + 6.43 [35]. To calculate the MELD-Na score, we used the formula: MELD-Na = MELD − Na − [0.025 × MELD × (140-serum Na)] +140 [36].

Moreover, we evaluated the fulfillment of KCC based on clinical and laboratory data from the first day of hospitalization. In ALF after paracetamol ingestion, an arterial pH < 7.30 after bolus infusion or PT > 100 s (INR > 6.5) and creatinine level over 3.5 mg/dL and grade III or IV HE is associated with an indication of LT. In ALF with other causes (non-paracetamol), PT > 100 s (INR > 6.5) or three of the following criteria (non-AB viral hepatitis, toxic or unknown cause, more than 7 days from the onset of jaundice to the start of the HE, age under 7 years or over 40 years, PT > 50 s, INR > 3.5 or TB > 17.4 mg/dL) are associated with an indication of LT [37].

For nine patients with ALF presentation in WD, we calculated the Nazer prognostic index and Wilson index for predicting mortality on the first day of hospitalization. The Nazer index is calculated based on the serum level of AST, bilirubin, and PT and a value of 7 points or higher indicates the need for LT for survival [38]. The Wilson index for predicting mortality is based on the serum level of TB, INR, AST, albumin, and WBC. A cut-off score of 11 points was identified for death without LT [39].

Our analysis did not include the data from the following days of hospitalization as many patients received fresh frozen plasma during their ALF management, and the scores could be biased by this or other treatments received.

We included all data in a Microsoft Office Excel database. For the statistical analysis, we used Statistica version 13 (TIBCO Software Inc., Palo Alto, CA, USA, 2018). We have used descriptive statistics for continuous distribution variables (means and standard deviations) and categorical variables (frequencies and percentages). We have used the t-Student test, Mann–Whitney test (for PELD, MELD, and MELD-Na scores), and Pearson Chi-square (for correlation between qualitative variables). We have calculated the sensitivity, specificity, PPV, NPV, and accuracy for KCC criteria and PELD, MELD, and MELD-Na scores. Discrimination, a model’s ability to distinguish patients with different disease outcomes, was assessed using the receiver operating characteristic (ROC) curves. We calculated the area under ROC curve (AUROC) values, confidence intervals, and cut-off values for PELD, MELD, MELD-Na scores, INR, and TB. We used the EasyROC software available online [40]. We selected the cut-off values using the Youden Index, the maximum potential effectiveness index for a test’s ability to classify a disease’s outcome. With an AUROC over 0.7, the test is considered clinically useful, and an AUROC over 0.8 is considered excellent diagnostic accuracy. However, the test does not correctly predict the outcome for 20% of cases in the last situation [21]. We analyzed the performance of PELD, MELD, MELD-Na scores, and KCC in predicting the outcome of ALF function to the age of the patients and ALF etiology. The results were considered statistically significant at values of *p *< 0.05.

## 3. Results

We included 161 children with ALF in the study, aged between 2 weeks and 17 years and 11 months (mean age 7.66 ± 8.19 years); 88 females (54.66%). According to the age groups, there were 42 infants (26.09%), 67 children (41.61%), and 52 teenagers (32.30%).

The etiology of ALF was different by age group and included toxic causes (64 patients, 39.75%, drug-induced in 51 patients, and mushroom poisoning in 13 patients), infectious diseases (41 patients, 25.45%), metabolic causes (27 patients, 16.77%: IEM in 18 patients and WD in 9 patients), and autoimmune diseases (15 patients, 9.32%). In 14 patients (8.69%), the etiology remained unknown.

A good outcome, as survivors with native liver after supportive treatment, was in 106 patients (65.84%), and 55 children (34.16%) had unfavorable evolution (three patients with emergency LT and 52 with fatal outcome, 32.30%). Emergency LT was performed in two girls with fulminant WD (four days after diagnosis) and one infant with metabolic disease (one week after diagnosis) that otherwise would not survive.

The demographic data, and clinical and laboratory parameters of our patients are presented in Table 1, based on the outcome of ALF. Patients with poor outcomes were younger, presented on their first day of hospitalization with higher bilirubin and INR and lower albumin and hemoglobin. They presented more frequently HE, metabolic acidosis, and kidney injury.

The PELD score was calculated for 109 patients with age under 12 years, and the mean value was 16.58 ± 11.80. For 52 patients between 12 and 18 years, the MELD and MELD-Na score was calculated, and the mean value was 24.33 ± 12.64 and 23.84 ± 10.24, respectively. The PELD score was significantly higher (21.04 ± 13.28) in patients with poor outcomes than survivors (13.99 ± 8.57; *p* = 0.002282). The teenagers with unfavorable evolution had a higher MELD and MELD-Na score than survivors with native liver (36.20 ± 13.44 and 33.07 ± 8.29 vs. 19.51 ± 8.57 and 20.08 ± 8.47; *p* < 0.00001).

The PELD score in neonates and infants did not differ based on the ALF evolution: 21.99 ± 14.50 in those with poor outcomes vs. 19.07 ± 12.72 (*p* = 0.474096) in survivors. However, in children aged 1 to 12 years, the PELD score was significantly higher in those with poor outcomes (19.62 ± 11.52 vs. 11.61 ± 7.61, *p* = 0.002415).

In Table 2, the PELD, MELD, and MELD-Na scores in different causes of ALF are presented based on the outcomes. In toxic causes, WD, and ALF’s unknown causes, these scores were significantly higher in patients with poor outcomes. There was no difference for infectious, IEM, or autoimmune causes of ALF.

**Figure 1:**
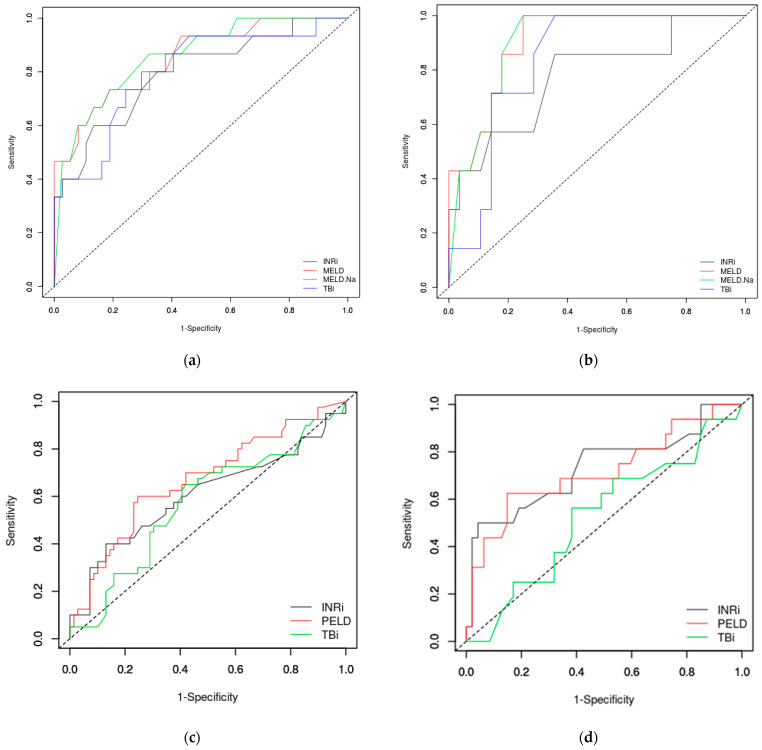
ROC curves in predicting the fatal outcome in ALF: teenagers (**a**), toxic causes (**b**), children up to 12 years old (**c**) and children from one to 12 years (infants and neonates excluded) (**d**). MELD— Model for End-Stage Liver Disease; MELD-Na—Model for End-Stage Liver Disease including sodium level; PELD—Pediatric End-Stage Liver Disease; INR—International Normalized Ratio; TB—total bilirubin.

We analyzed the performance of the MELD and MELD-Na scores compared to INR and TB serum levels in discriminating the teenagers with poor outcomes from the survivors using ROC curves (Figure 1a,b). In teenagers, INR (using a cut-off of 3) had an AUROC of 0.7946, TB (cut-off 2.3 mg/dL) had an AUROC of 0.7991, but MELD and MELD-Na score (both at a cut-off of 28) performed better with AUROC 0.8495 and 0.8576, respectively. In those with toxic causes, MELD and MELD-Na scores predict the fatal outcome better but at a lower cut-off (Table 3).

In children up to 12 years old, the PELD score performed lower (AUROC 0.6686) than the MELD score in teenagers, but better than INR or TB in predicting the fatal outcome in ALF. If we excluded the neonates and infants, the PELD score has better performance (AUROC 0.7181), but also INR over 3 mg/dL performed well (AUROC 0.7335) (Table 3 and Figure 1c,d). In neonates and infants, PELD score, INR and TB had a low accuracy with an AUROC under or close to 0.6 (Table 3). 

In our cohort, KCC proved to have a sensibility of 29.09%, specificity of 94.34%, PPV of 72.73%, and NPV of 71.95%. The accuracy was 72.05%. If we analyzed the KCC criteria in children over one year of age (as analyzed in the original cohort for KCC), the performance increased: sensibility of 32.26%, specificity of 98.81%, PPV of 90.91%, NPV of 79.17%, and accuracy of 80.87%. The best performance was in teenagers with a specificity of 100%, PPV of 100%, NPV of 82.22%, and accuracy of 84.61% (Table 3).

Analyzing KCC for the patients with toxic ALF (64 children), 4 patients with fatal evolution and no survivors had positive KCC, but 7 patients with poor outcomes did not fulfill the KCC. In children with metabolic diseases (27 patients), 8 children with fatal evolution and 3 survivors had positive KCC. Yet, there were 10 patients with unfavorable evolution without having KCC. In ALF after infections (41 patients), none of those 15 patients with fatal outcomes had positive KCC. At the same time, these criteria were present in three survivors. In patients with ALF due to autoimmune diseases (15 patients), only one patient died without positive KCC. Ten children with ALF of unknown cause had a fatal evolution, and only four had positive KCC. Those four who survived with native liver were without positive KCC. Based on all these data, KCC proved to have a specificity between 66.67% in metabolic ALF and 100% in toxic, autoimmune, and unknown causes. Overall, KCC had the best performance in toxic causes of ALF (Table 3).

In those nine children with WD, we calculated the Nazer prognostic index and Wilson index for predicting mortality [38,39]. Six patients scored seven or higher for Nazer prognostic score: five teenagers with unfavorable evolution of ALF (two girls with emergency LT and three who did not survive) and one child who survived. The other three survivors scored under seven. Based on these data, the accuracy of Nazer score for predicting the unfavorable outcome was 88.9%, with a sensitivity of 100%, specificity of 75%, PPV of 83.3%, and NPV of 100%. Regarding the Wilson index for predicting mortality, 5 patients scored over 11, all with unfavorable evolution. Based on these data, the accuracy of the Wilson index was 100%, with a sensitivity, specificity, PPV, and NPV of 100%, proving to be better than the previously used Nazer prognostic index. In WD patients, MELD/PELD scores were higher in patients with poor outcomes (45.80 ± 8.93 vs. 13.37 ± 6.20, *p* = 0.000473). Those with unfavorable outcome were with higher serum level of TB (32.90 ± 18.30 mg/dL vs. 1.73 ± 0.86 mg/dL, *p* = 0.012), DB (27.36 ± 16.05 mg/dL vs. 1.33 ± 0.57 mg/dL, *p* = 0.015), INR (6.14 ± 2.83 vs. 2.62 ± 0.58, *p* = 0.046), and lower albumin level (2.58 ± 0.52 g/dL vs. 3.43 ± 0.17 g/dL, *p* = 0.017). There were no significant differences in serum level of transaminases, ceruloplasmin, or hemoglobin in those with poor outcomes than survivors. Analyzing KCC only in WD patients, from five with unfavorable outcomes, four had KCC. None of the survivors had positive KCC. Considering the small number of patients, the specificity of KCC was 100%, sensitivity 80%, PPV 100%, NPV 80%, and the accuracy was 88.9%.

## 4. Discussion

ALF is rare in children but is characterized by a high severity and risk for a fatal outcome if proper treatment or sometimes emergency LT is impossible. It is essential in such cases to have accurate predictive tools to discriminate patients with high risk to include them early on the emergency LT list and identify those that will recover spontaneously or with proper specific treatment [3,19,41].

In adults, many parameters were evaluated for their prognostic value in ALF: peak TB level [17,42,43], transaminases [8,9,42], INR or PT [9,17,43,44,45], presence of grade III/IV HE [1,29,43,44,45], more than seven days to the onset of HE [9,45], age [8,17,45], serum creatinine [45], thrombocytopenia as a marker of multiple organ failure [3,46], high ammonia [8,17,26,27], high plasma lactate [17,47], actin-free Gc globulin [26,48,49,50,51,52], and serum phosphate [26,53,54]. Galectine-9 as a marker of liver injury was evaluated as a prognostic factor in DILI [55]. Other new parameters studied in adults with ALF were IL6, IL10, IL8, soluble CD154, and apoptosis marker M30 of the cytokeratin 18 [1,17,54,56]. The most important problem regarding these parameters is the lack of availability of these measurements in any hospital and the need for quick results to help an early decision.

Studies on different clinical and laboratory parameters in ALF in children found that high TB level [3,44,57], increased INR [1,3,44,57], lower transaminases [58], young age [12,57,59], and encephalopathy [3,44,57] are prognostic factors for those who will need LT. Our team recently published a study on the role of Gc-globulin as a prognostic marker in children with ALF [25].

There are many predictive models on the need for LT in ALF, but none is universally accepted in adults and children [28]. The ideal predictive model should be based on standard clinical and laboratory parameters, easy to measure in any hospital, and dynamic analysis. Data on a particular moment may have a limited value, and the risk should be evaluated during disease evolution [21,31,58,60,61]. Predictive models need to be studied further as improvements should be made to consider the severity of liver necrosis and the possibility of liver regeneration [26]. Moreover, they need to include the age and etiology of ALF [1,62].

The MELD and PELD scores were designed to assess the risk of mortality and need of LT in chronic liver disease, but their usefulness in ALF was assessed more and more in the last years [1,45,63].

KCC are used for evaluating the need for LT in ALF. Their presence predicts mortality without LT, but their absence is not necessarily associated with survival [18,64]. KCC are used mainly in adults and were not validated in children [37]. KCC may differ in accuracy in children than adults as HE is challenging to assess, and the etiology may be different or even unknown in many children. Moreover, in the original study, none of those 29 children included were under 1 year [37]. KCC may not successfully allow a correct decision for LT as sometimes predict the need of LT in more children than they would need [62,65].

Based on age, HE, and serum level of factor V, Clichy criteria proved low sensitivity [24]. It was reported to have a PPV of 0.82 and NPV of 0.98, but the cohort was made of adults with fulminant HBV, so it is difficult to generalize these results [21]. We did not evaluate Clichy criteria in our cohort, as we did not have data regarding the factor V level for all subjects.

Our study aimed to analyze the possible role of PELD, MELD scores, and KCC in a large cohort of children with ALF from two important hospitals in our country. Our cohort is uniformly distributed on age and includes patients with various causes of ALF (toxic, infections, autoimmune disorders, and metabolic diseases). We considered the LT patients as unfavorable outcomes because they probably would not survive without LT. One of the problems in all modeling is the end-point. All models have limitations as all studies suggest that TB, INR, and HE may predict poor outcomes. If the decision to perform LT is based on these factors, the results of the predicting models may be biased [61]. As Schneider et al. commented, if LT is excluded from the analysis, the PELD score could not be significant in predicting poor outcomes [16]. In our study, only three patients were with emergency LT.

The PELD and MELD scores should be used cautiously in ALF in children as there is a high heterogeneity with various etiologies, different ages. Moreover, in adults, there is a problem regarding the characteristics of hyperacute patients (associated with a good chance for spontaneously recovering, even with high INR and encephalopathy) and subacute patients (associated with poor prognosis) [31]. The PELD score was validated for the need for LT in chronic diseases to predict 3-month pretransplant mortality [3,66]. It includes growth failure and serum albumin level that are not usually associated with ALF [3].

The PELD score at different cut-offs was studied and proved to have better sensitivity and specificity than TB, INR, or HE in predicting poor outcomes in ALF. Sanchez and D’Agostino reported that, at a cut-off of 33, the PELD score had a better sensitivity and specificity than KCC in a cohort of 40 children, most of them with HAV infection [12]. Other studies found significant a lower cut-off for PELD, at 27 or 28 [58,67].

Yantorno et al. [68] reported that a MELD score over 30 is superior to KCC and Clichy, with a PPV of 91% and an NPV of 100%. In this study, the adolescents were excluded. Many meta-analyses were published assessing the performance of MELD score and KCC in ALF in adults. Based on 23 studies, including 2153 patients [69], MELD score and KCC proved to be comparable in accuracy, but KCC has a lower sensitivity and MELD has a lower specificity. The presence of HE, subjectively included in KCC, weakens the performance of KCC compared to MELD [69]. Another meta-analysis assessed KCC in non-acetaminophen and proved the higher specificity of KCC [61]. A systematic review based on 14 studies calculated for KCC a specificity of 94.6%, with lower sensitivity (58%) and an AUC of 0.91 [54]. Based on etiology, KCC had a 70–88% diagnostic accuracy in acetaminophen-induced ALF and 55 to 92% in non-acetaminophen ALF [26]. In non-acetaminophen ALF, up to 70% died without KCC, and 21% of those with KCC would survive without LT [26]. In a cohort of 522 children with non-paracetamol ALF, the KCC had a lower sensitivity than in adults with similar specificity [37].

In our study, the MELD score, at a cut-off of 28, proved to have an 81.1% specificity and 73.3% sensitivity, similar to that reported by Sanchez, D’Agostino [12] and Yantorno [68], and better than the admission day results reported by Rajanayagam [58]. Using the peak values in Rajanayagam’s study [58], the MELD score had similar results as in our study. We did not analyze the dynamic of scores during evolution, but this would be the best approach if some treatment effects were considered. It was proved that serial determination of scores is superior to a single measurement at admission [58,60].

Analyzing the performance of the MELD score by diagnosis for toxic causes of ALF, we proved that the MELD score at admission is an excellent predictor of poor outcome, having excellent sensitivity but lower specificity.

In our cohort, in children under 12 years, the PELD score performed lower. Excluding the infants, the performance increased, but remained lower than the MELD score. Interestingly, INR > 3 was a slightly better predictor of poor outcome than the PELD score in this subgroup of patients.

In our study, KCC demonstrates a high specificity but lower sensitivity, as was described in a previously published meta-analysis [54,61,69]. The accuracy was not as high as in adult studies. If we excluded the infants from the analysis, the performance of KCC improved. In our cohort, the best performance of KCC was shown in teenagers.

HE is an important prognostic factor in adults (included in KCC or Clichy criteria, but not in the MELD score). However, it is challenging to diagnose HE in children, and it could be rapidly progressive [1]. In the PALF study group, mortality was higher in children presenting with grade III and IV HE or rapid progression after admission [70]. In our study, the patients with poor outcomes presented on admission day more frequently HE than the survivors. Moreover, metabolic acidosis and acute kidney injury were more frequent in children with unfavorable outcomes.

Studies have tried to improve the MELD score and KCC by adding other parameters. However, including the lactate and phosphate level did not bring a clear advantage, for although the sensitivity of KCC was improved, the specificity was reduced [53,71]. Adding serum sodium to the MELD score increased the predictive value [36]; however, there were no significant differences in our study when the MELD-Na score was calculated.

There are more criteria to predict the fatal outcome in ALF due to mushroom poisoning, but none are universally accepted [72]. Besides KCC, Ganzert’s (a decrease in prothrombin index under 25% of normal between the 3rd and 10th day after ingestion associated with a serum creatinine over 1.2 mg/dL) [73] and Escudie’s criteria (interval between ingestion and diarrhea under 8 h or a decrease in prothrombin index under 10% of normal, INR > 6, at four or more days after ingestion) [74] have also proved to be helpful in ALF due to mushroom poisoning. Ganzert proved that the prothrombin index and serum creatinine were significantly better predictors than serum bilirubin and ALT [73]. In our previous works, in children mostly with ALF due to mushroom poisoning, serum creatinine and HE presence were found to predict mortality independently [75]. Moreover, in a study including 17 children with ALF due to mushroom poisoning, a PELD score over 20 had a 100% sensitivity and specificity to predict the fatal outcome, slightly better than KCC’s 77.7% sensitivity and 100% specificity [76]. The present study, including a larger cohort of pediatric ALF patients, did not analyze mushroom poisoning separately because the number of patients with this cause of ALF decreased in the last years in our country [77]. Instead, we analyzed all toxic causes of ALF in 64 patients. The MELD score (at a cut-off of 24) had an excellent power of discrimination. The MELD score performed better than INR over 2.5 or TB. Moreover, in children under 12 years old, the PELD score was significantly higher in those with fatal outcomes than in survivors. KCC performed similarly in these patients with 100% specificity and lower sensitivity. The accuracy of KCC was similar to the MELD score. For other etiologies of ALF in our cohort, KCC had lower accuracy, excepting autoimmune disorders (there was only one case with fatal evolution but without fulfilling the KCC).

The PELD and MELD scores do not consider comorbidities, such as infections and sepsis, which may increase mortality [60,78]. Some authors tried to evaluate the ICU scores due to these aspects. Acute Physiology and Chronic Health Evaluation (APACHE II) [56,79,80], Acute Liver Failure Study Group (ALFSG) index [56,81], Sequential Organ Failure Assessment (SOFA) [41,80], pediatric Chronic LIver Failure Sequential Organ Failure Assessment (pCLIF-SOFA) [29], Pediatric RISk of Mortality (PRISM) [30], Pediatric Index of Mortality (PIM) [82], Acute Liver Failure Early Dynamic (ALFED) model [11,83], and Simplified Acute Physiology Score III (SAPS III) [17,84] were analyzed in various studies. Still, the heterogeneity of the cohorts and results make it difficult to support that they are a better predictor than MELD or KCC in ALF patients [30].

LIU (Liver Injury Units) based on peak levels of bilirubin, PT, and ammonia proved to be strongly predictive in ALF in children. LIU, based on admission levels, has moderate predictive strength [20]. LIU may be used daily to predict the possible outcome anytime during hospitalization [85].

In WD patients, the Wilson index for predicting mortality could predict very well the need for an emergency LT [38,86]. In our study, the accuracy, sensitivity, and specificity were 100%, better than the Nazer prognostic index performance. Moreover, KCC had a specificity of 100%, but a lower accuracy. The MELD/PELD score was higher in those with fatal outcomes (over 30) than survivors (under 20). Our data support the need to evaluate the Wilson index for predicting mortality on the admission day of a child with WD and ALF to early referral to an LT center [86].

## 5. Conclusions

ALF is a severe disease in children, and there are clinical and laboratory parameters or scores that can help us predict poor outcomes. Children with unfavorable evolution are younger, and present with higher TB and INR, and lower albumin and hemoglobin. They present HE, metabolic acidosis, and acute kidney injury more frequently than survivors. The PELD and MELD scores, and KCC and Wilson indexes, proved helpful in children with ALF, having a good accuracy in discriminating patients who need emergency LT. The MELD score had the best performance in teenagers with ALF of toxic cause, and the PELD score had a lower performance than the MELD score. The PELD score and KCC had low accuracy for predicting fatal evolution in neonates and infants. KCC had the highest accuracy in teenagers and ALF of toxic causes. The MELD score and KCC had a similar accuracy, but MELD had a higher sensitivity, and KCC a higher specificity. Besides the improvements in supportive measures, mortality in ALF in children was still high, and the emergency LT represents the only hope for some of them. For this reason, finding the best predicting score in ALF, and early referral of the children to a specialized center, are the most critical issues to improve survival.

## Figures and Tables

**Table 1:** Demographic data, clinical and laboratory parameters, and prognostic scores in children with ALF based on the disease outcome.

Variable	Survivors with Native Liver under Medical Treatment (*n* = 106)	Poor Outcome (LT or Deceased) (*n* = 55)	*p*-Value
**Age (years)**	**8.53 ± 5.91**	**5.51 ± 6.43**	**0.003304**
Males (*n*, %)	45 (42.45%)	28 (50.91%)	0.30670
AST (UI/L)	1 332.02 ± 2 057.03	1 028.38 ± 1 779.19	0.354363
ALT (UI/L)	1 051.47 ± 1 305.89	699.35 ± 1 229.77	0.099954
**TB (mg/dL)**	**4.40 ± 5.45**	**9.27 ± 12.30**	**0.000663**
**DB (mg/dL)**	**3.26 ± 4.41**	**7.04 ± 10.05**	**0.001158**
**INR**	**2.44 ± 1.21**	**3.76 ± 2.78**	**0.000046**
**Albumin (mg/dL)**	**3.29 ± 0.48**	**2.97 ± 0.71**	**0.000732**
**Hemoglobin (g/dL)**	**10.79 ± 2.26**	**9.36 ± 2.34**	**0.000341**
White blood cells (/mm^3^)	14 422 ± 21 178	14 126 ± 8 990	0.921381
Platelets (/mm^3^)	198 490 ± 106 904	207 418 ± 166 127	0.680128
Ceruloplasmin (mg/dL) *	10.175 ± 5.863	7.75 ± 4.179	0.52570
**Encephalopathy (*n*, %)**	**17 (16.04%)**	**28 (50.91%)**	**<0.00001**
**Metabolic acidosis (*n*, %)**	**58 (54.72%)**	**40 (72.73%)**	**0.02637**
**Acute kidney injury (*n*, %)**	**13 (12.26%)**	**27 (49.09%)**	**<0.00001**
Renal replacement (*n*, %)	11 (10.38%)	10 (18.18%)	0.16317
**PELD (*n* = 109)**	**13.99 ± 10.07**	**21.04 ± 13.28**	**0.002282**
**MELD (*n* = 52)**	**19.51 ± 8.57**	**36.20 ± 13.44**	**0.000002**
**MELD-Na (*n* = 52)**	**20.08 ± 8.47**	**33.07 ± 8.29**	**0.000006**
**KCC (*n*, %)**	**6 (5.66%)**	**16 (29.09%)**	**0.0004**

LT—liver transplantation; AST—aspartate aminotransferase; ALT—alanine aminotransferase; TB—total bilirubin; DB—direct bilirubin; INR—International normalized ratio; PELD—Pediatric End-Stage Liver Disease; MELD—Model for End-Stage Liver Disease; MELD-Na—Model for End-Stage Liver Disease including sodium level; KCC—King’s College Criteria. Continuous parameters are represented as mean ± standard deviation and categorical parameters as number and percentage. * Serum level of ceruloplasmin was evaluated only for patients with Wilson’s Disease.

**Table 2:** The PELD, MELD, MELD-Na scores function to the etiology of acute liver failure.

	Survivors with Native Liver under Medical Treatment	Poor Outcome (LT or Deceased)	*p*-Value
**Toxic causes**			
**PELD**	**9.90 ± 7.16**	**25.32 ± 17.16**	**0.003192**
**MELD**	**18.36 ± 9.09**	**37.28 ± 10.73**	**0.000037**
**MELD-Na**	**18.82 ± 8.96**	**34.28 ± 6.02**	**0.000142**
**Infectious causes**			
PELD	13.63 ± 7.80	15.52 ± 9.61	0.546069
MELD	17 ± 4.24	26 ± 0	0.333333
MELD-Na	21 ± 7.07	26 ± 0	0.666667
**Autoimmune causes**			
PELD	15.06 ± 6.12	14.50 ± 0	0.9333
MELD	23.50 ± 6.89		
MELD-Na	24.17 ± 6.55		
**Metabolic causes**			
IEM—PELD	31.92 ± 14.06	25.90 ± 16.92	0.491631
WD—MELD	20 ± 0	45.80 ± 8.93	0.057692
**WD—MELD-Na**	**21 ± 0**	**39 ± 2.24**	**0.001826**
**Unknown etiology**			
**PELD**	**5.467 ± 1.955**	**23.544 ± 8.898**	**0.006922**
MELD	21 ± 0	19 ± 0	
MELD-Na	24 ± 0	19 ± 0	

PELD—Pediatric End-Stage Liver Disease; MELD—Model for End-Stage Liver Disease; MELD-Na—Model for End-Stage Liver Disease including sodium level; LT—liver transplantation, IEM—inborn errors of metabolism; WD—Wilson’s disease.

**Table 3:** Performances of MELD, MELD-Na, and PELD scores, INR, TB serum level, and KCC in predicting the fatal outcome in ALF.

Parameter	Cut-Off	Sensitivity	Specificity	PPV	NPV	AUROC
**Teenagers (*n* = 52)**						
**INR**	3	60%	86.5%	64.3%	84.2%	0.7946
**TB**	2.3 mg/dL	80%	70.3%	52.2%	89.7%	0.7991
**MELD**	28	73.3%	81.1%	61.1%	88.2%	0.8495
**MELD-Na**	28	73.3%	81.1%	61.1%	88.2%	0.8576
**Teenagers with ALF of toxic causes (*n* = 35)**						
**INR**	2.5	85.7%	64.3%	37.5%	94.7%	0.7781
**TB**	1.3 mg/dL	100%	64.3%	41.2%	100%	0.8367
**MELD**	24	100%	75%	50%	100%	0.9056
**MELD-Na**	26	100%	75%	50%	100%	0.9031
**Children under 12 years old (*n* = 109)**						
**INR**	3	40%	87%	64%	71.4%	0.6045
**TB**	3.4 mg/dL	65%	58%	47.3%	74.1%	0.5725
**PELD**	18.9	60%	75.4%	58.5%	76.5%	0.6686
**Children under 12 years old—excluding neonates and infants (*n* = 63)**						
**INR**	3	50%	95.7%	80%	84.9%	0.7334
**TB**	4 mg/dL	56.2%	61.7%	33.3%	80.6%	0.5286
**PELD**	19.5	62.5%	85.1%	58.8%	87%	0.7181
**Neonates and infants (*n* = 46)**						
**INR**	10	12.5%	100%	100%	51.2%	0.4896
**TB**	6.6 mg/dL	54.2%	77.3%	72.2%	60.7%	0.6146
**PELD**	12.9	75%	40.9%	58.1%	60%	0.5511
						**Accuracy**
**KCC based on age**						
**Neonates and infants (*n* = 46)**		25.00%	77.27%	54.55%	48.57%	50%
**Children (*n* = 63)**		18.75%	97.87%	75%	77.97%	77.78%
**Teenagers (*n* = 52)**		46.67%	100%	100%	82.22%	84.61%
**KCC based on ALF etiology**						
**Toxic causes (*n* = 64)**		36.36%	100%	100%	88.33%	89.06%
**Metabolic (*n* = 27)**		44.44%	66.67%	72.73%	37.50%	51.85%
**Infectious (*n* = 41)**		0%	88.46%	0%	60.53%	56.10%
**Autoimmune (*n* = 15)**		0%	100%	0%	93.33%	93.33%
**Unknown (*n* = 14)**		40%	100%	100%	40%	57.14%

MELD—Model for End-Stage Liver Disease; MELD-Na—Model for End-Stage Liver Disease including sodium level; PELD—Pediatric End-Stage Liver Disease; INR—International Normalized Ratio; TB—total bilirubin; KKC—King’s College Criteria; PPV—positive predictive value; NPV—negative predictive value; AUROC—Area Under the Receiver Operating Characteristic.

## Data Availability

Data are available on request from the corresponding author.
